# hnRNP A1: The Swiss Army Knife of Gene Expression

**DOI:** 10.3390/ijms140918999

**Published:** 2013-09-16

**Authors:** Jacques Jean-Philippe, Sean Paz, Massimo Caputi

**Affiliations:** Charles E. Schmidt College of Medicine, Florida Atlantic University, 777 Glades Rd, Boca Raton, FL 33431, USA; E-Mails: jjeanph3@fau.edu (J.J.-P.); cprsean@gmail.com (S.P.)

**Keywords:** hnRNP, mRNA, transcription, splicing, translation, telomere, miRNA

## Abstract

Eukaryotic cells express a large variety of RNA binding proteins (RBPs), with diverse affinities and specificities towards target RNAs. These proteins play a crucial role in almost every aspect of RNA biogenesis, expression and function. The heterogeneous nuclear ribonucleoproteins (hnRNPs) are a complex and diverse family of RNA binding proteins. hnRNPs display multiple functions in the processing of heterogeneous nuclear RNAs into mature messenger RNAs. hnRNP A1 is one of the most abundant and ubiquitously expressed members of this protein family. hnRNP A1 plays multiple roles in gene expression by regulating major steps in the processing of nascent RNA transcripts. The transcription, splicing, stability, export through nuclear pores and translation of cellular and viral transcripts are all mechanisms modulated by this protein. The diverse functions played by hnRNP A1 are not limited to mRNA biogenesis, but extend to the processing of microRNAs, telomere maintenance and the regulation of transcription factor activity. Genomic approaches have recently uncovered the extent of hnRNP A1 roles in the development and differentiation of living organisms. The aim of this review is to highlight recent developments in the study of this protein and to describe its functions in cellular and viral gene expression and its role in human pathologies.

## 1. Introduction

In the past decade, advances in genomic research have highlighted the fundamental role that post-transcriptional events play in the regulation of gene expression. RNA binding proteins (RBPs) control and regulate the various steps of the maturation of nascent transcripts. RBPs modulate the capping, splicing, polyadenylation, nuclear export, stability rates and translation of cellular messenger RNAs (mRNAs) by binding specific sequences or secondary structures within the transcripts. Heterogeneous nuclear ribonucleoproteins (hnRNPs) are among the best-studied and largest families of RBPs.

Historically, hnRNPs have been defined as proteins that associate with nascent transcripts (namely, pre-mRNAs or hnRNAs), precursors of the functional, protein coding mRNAs [1]. Initial studies indicated that nascent RNA polymerase II (RNAPII) transcripts were packaged with six “core” hnRNP proteins (A1, A2, B1, B2, C1 and C2) in a bead on a string structure that resembles the organization of DNA nucleosomes [2]. However, the biochemical and functional characterization of over 20 individual hnRNPs, with distinct RNA binding specificities, led to a complex model characterized by dynamic hnRNP complexes, regulating the processing and expression of the nascent transcripts.

The hnRNP family is composed of at least 20 abundant, major hnRNPs and other less abundant, minor members. The more abundant family members, named A through U, have molecular weights ranging from 34 kDa to 120 kDa [1,3]. hnRNPs share a modular structure consisting of one or more RNA-binding domains (RBDs). RNA recognition motifs (RRMs) are the most common RBDs found in hnRNPs; these globular domains are highly conserved and are found in several other protein families. RRMs are approximately 90 amino acids-long and have the capacity to participate in both general and specific interactions with nucleic acids [4]. Other common RBDs that contribute to the binding specificity of the single hnRNPs are: RGG boxes, which are repeats of Arg-Gly-Gly with interspersed aromatic amino acids and K-homology (KH) domains, characterized by evolutionarily conserved sequence of around 70 amino acids [5,6]. In addition to RBDs, hnRNPs contain auxiliary domains, such as glycine-rich, acidic or proline-rich domains, which mediate protein-protein interactions, subcellular localization and the functional specificity of single proteins [7]. Biochemical and bioinformatics approaches have shown that hnRNPs play both a constitutive role, as RNA packaging proteins, in RNA biogenesis, and a regulatory role by binding specific RNA sequences and by interacting with other regulatory factors; such functions are dependent on both RBDs and auxiliary domains. hnRNPs undergo several post-translational modifications, including phosphorylation, SUMOylation, ubiquitination and methylation, which regulate their activities. Although hnRNPs generally have a diffuse nuclear distribution, many remain bound to the mRNA as it is transported through nuclear pores, associates with the ribosome or is sequestered in specific cellular compartments. Hence, post-translational modifications may modulate hnRNPs activity by altering their localization, RNA binding specificity and interaction with other cellular factors [8].

The complex formed by hnRNPs and nascent transcripts is highly dynamic, and it is remodeled through the loss or acquisition of hnRNPs and other proteins. The interaction of hnRNPs, as well as other RNA-binding proteins with a given transcript creates what has been termed the “mRNP code” [9], which regulates the maturation and expression of eukaryotic genes. Variation in the relative amount or modification in the activity of hnRNPs leads to changes in the processing, localization, stability and translation of the transcripts. Immunohistochemical data and a vast array of gene expression studies [10] revealed that hnRNPs are ubiquitously expressed in all tissue types to varying abundance levels, and their relative stoichiometry, across cell types, is not fixed. This suggests a role for this protein family as master regulators of gene expression. More recently, genome-wide analysis of cellular transcripts provided evidence of the combinatorial role of hnRNPs on global cellular gene expression, the regulation of cellular differentiation and the response to physiological stimuli [11].

Our understanding of the functions of single hnRNPs is highly uneven. Few hnRNPs have been widely studied, while little information is available about the structure and function of the rest. The following sections will review our current understanding of hnRNP A1, a member of the A/B subfamily and arguably the best known among the hnRNPs. We will highlight the multifunctional nature of this protein, describing its role in several cellular processes.

## 2. The hnRNP A/B Family

hnRNP A1 is a member of the hnRNP A/B subfamily, which comprises four paralogues, A1, A2/B1, A3 and A0, and two more distantly related proteins, B2 and AB [12,13]. Initial studies indicated that members of the hnRNP A/B and hnRNP C subfamilies package nascent transcripts in a non-sequence-specific manner, constituting the core hnRNP particle [2,14]. Subsequent analysis of hnRNPs A/B binding specificities have shown that these proteins have also distinct and specific preferences for a specific subset of RNA sequences [15–18]. The molecular basis of the duality in the RNA-binding ability of the hnRNP A/B family members is not well understood. It is conceivable that both the multiple RNA binding domains present within hnRNPs A/B and the local concentration of these proteins within specific subcellular compartments might contribute to both general scaffolding and sequence-specific RNA binding activities. The hnRNPs A/B sequence-specific binding activity and their ability to interact with other cellular factors allow for a multiplicity of functions in pre-mRNA splicing, mRNA trafficking, translation, microRNA (miRNA) processing and telomerase maintenance.

## 3. Structural Features of hnRNP A1

**Figure 1:**
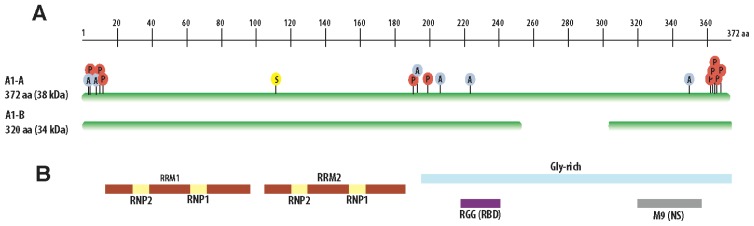
Structural features h nuclear ribonucleoprotein (hnRNP) A1. (**A**) Schematic map of the two hnRNP A1 isoforms. Acetylation (A), phosphorylation (P) and SUMOylation (S) sites are labeled; (**B**) Schematic representation of hnRNP A1 structural and functional domains.

hnRNP A1 is one of the most abundant nuclear proteins, rivaling histones in its amount, and although multiple alternatively spliced transcript variants have been predicted for this gene, only two transcripts have been validated experimentally: A1-B, the full-length isoform of 372 amino acids (38 kDa), and A1-A, the shorter variant, missing residues 253 to 303 (320 aa, 34 kDa), which, in most tissues, is over 20-times more abundant than the full-length protein. Structurally, hnRNP A1 can be divided in an *N*-terminal domain that contains two closely-related RRM domains, followed by a highly flexible glycine-rich (Gly-rich) *C*-terminal region, which contains an RGG box RNA binding domain and a nuclear targeting sequence, termed M9 (Figure 1) [19]. Although the secondary structure of the full-length hnRNP A1 has not been confirmed experimentally, the *N*-terminal, termed the UP1 fragment, has been extensively studied by Z-ray crystallography and NMR spectroscopy [20]. Several high-resolution crystal structures of the two tandem RRMs of hnRNP A1 have been solved both in their free form and bound to telomeric DNA repeats at a resolution of 1 Å [21–25]. Eukaryotic RRMs are composed of four β-strands sheets and two α-helices positioned in a β1α1β2β3α2β4 structure and characterized by two highly conserved sequences, RNP1 (octamer) and RNP2 (examer), which are located about 30 residues apart [4]. The conserved RNP1 and RNP2 sequences are juxtaposed on the β3 and β1 strands and make direct contact with the RNA. The β-sheet surface of the RRM constitutes a free plane that functions as a platform for RNA binding. The variable regions of the loops connecting the β-strands and the terminal regions of the RRM have been shown to be important determinants of RNA-binding specificity [26]. These regions differ in amino acid sequence among single hnRNP proteins, thus contributing to the differences in their RNA binding specificities. In spite of their similar sequences and overall structure, the two RRMs of hnRNP A1 are neither redundant nor functionally equivalent. It is unclear how these RRMs contribute to the RNA binding properties of hnRNP A1, since duplications, deletions or swapping of the RRMs differently affects the alternative splicing functions of hnRNP A1 [27].

The Gly-rich 124 amino acid *C*-terminal domain has been shown to have both RNA and protein binding properties [28]. The Gly-rich domain contains a motif characterized by closely spaced clusters of Arg-Gly-Gly tripeptide repeats with interspersed aromatic (Phe, Tyr) residues, named the RGG box, which is thought to confer RNA binding functions to this domain [29]. Although the precise contribution of the RGG box to the overall RNA binding activity of hnRNP A1 is not well understood, experimental data indicate that it is responsible for cooperative binding of hnRNP A1 to its target RNA [30,31]. The Gly-rich domain is also required for homologous and heterologous interactions between hnRNP A1, other hnRNPs and RNA binding proteins [32]. The complexity and number of these interactions underline hnRNP A1 functions in RNA biogenesis [28,33], export [34] and telomere regulation [35].

Downstream of the RGG box and within the Gly-rich domain of hnRNP A1 is located a 38-amino acid nucleo-cytoplasmic shuttling (NS) domain, called M9 [36]. Like many of the hnRNP proteins, hnRNP A1 is predominantly nuclear at steady-state, but can shuttle between the nucleus and the cytoplasm in response to specific signals [37,38]. The M9 domain is both necessary and sufficient to confer nuclear localization and does not bear resemblance to the classical nuclear localization signal (NLS). Interestingly, this domain also acts as a nuclear export signal, allowing export and cytoplasmic accumulation of hnRNP A1 in response to specific signals [34]. The nuclear shuttling activity of hnRNP A1 is mediated by the direct interaction of the M9 sequence with two transport receptors of the karyopherin-β family, Transportin 1 and 2 (*Trn1*, *Trn2*) [39–41].

The interaction of hnRNP A1 with nucleic acids was first observed in early attempts to identify eukaryotic DNA-binding proteins [42]. Initial studies showed that both the RRM and Gly-rich domains of hnRNP A1 are involved in binding single-stranded and double-stranded DNA sequences [29,43]. These interactions are dependent on salt concentration, temperature and the methylation state of residues within the Gly-rich domain [44,45]. The affinity of hnRNP A1 for RNA sequences was established shortly after the initial discovery of its DNA binding abilities. Specific RNA sequences recognized by hnRNP A1 were isolated utilizing pools of interacting oligos by a SELEX procedure [46]. These RNA sequences shared the common UAGGGA(U) motif. Subsequent studies showed a clear affinity of hnRNP A1 for AUUUA-rich sequences contained within the 3′-untranslated region (UTR) of several eukaryotic genes [47]. Later, a number of functional hnRNP A1 binding sites were identified in several transcripts characterized by the UAGA(G) motif [17,48–52].

Post-translational modification in hnRNP A1 includes methylation, phosphorylation and SUMOylation. Many arginine residues within the RGG box are sites for methylation, which act to modulate nucleic acids binding [45,53,54]. Several serine residues in both RRMs and the Gly-rich domain have been shown to be phosphorylated both *in vitro* and *in vivo* [55–59]. Protein kinase C (PKC) and Mitogen-Activated Protein Kinase (MAP)-Interacting Kinases (MNKs) have been shown to phosphorylate a series of serine residues and to reduce hnRNP A1’s ability to bind specific RNA sequences and to regulate its localization [38,55–57]. hnRNP A1 also includes a SUMOylation site within the second RRM and appears to be SUMOylated *in vivo*, although the functional significance of this modification is unknown [60].

The RNA, DNA and protein binding specificities of hnRNP A1 define several functions in eukaryotic RNA maturation, maintenance, expression and genome stability. In the following sections, we will describe recent advances in our understanding of the functions of this protein in global gene expression and cellular metabolism.

## 4. Transcriptional Functions of hnRNP A1

hnRNP A1 has been shown to associate with multiple promoter sequences and to participate in the regulation of transcriptional events, although the precise mechanism is unclear. Association of hnRNP A1 with the promoters of genes coding for thymidine kinase (TK) [61], γ-fibrinogen [62] and the vitamin D receptor [43] induces transcriptional repression, while it functions as an activator upon binding onto the ApoE [63] and, possibly, the protein kinase regulated by RNA (PKR) [64] promoters. The ability of hnRNP A1 to bind G-quadruplex DNA structures [65,66] could also facilitate transcription by destabilizing and unwinding the G-quadruplex structure at the promoter of several genes. G-quadruplex formations in the human KRas and c-myc promoters are located nearby binding sites for hnRNP A1 [67,68]. It is conceivable that hnRNP A1 binding could relax the quadruplex DNA structure, promoting transcription initiation. An alternative mechanism might involve the 7SK small nuclear ribonucleoprotein (7SK snRNP), a molecular scaffold containing the non-coding 7SK small nuclear RNA (7SK), which binds the cellular factors, HEXIM1, HEXIM2 and LARP7, enabling the sequestration and inhibition of the transcription elongation factor, P-TEFb [69]. hnRNP A1 has been shown to bind the 7SK RNA, promoting disassociation of P-TEFb from the 7SK snRNP and its assembly onto the active RNA polymerase II (RNAPII) transcription complex, thus activating gene transcription [70,71]. In addition to modulating transcriptional events through binding DNA and RNA sequences, hnRNP A1 can also directly regulate the activity of transcription factors through protein-protein interactions. hnRNP A1 has been shown to interact with the inhibitory subunit of NF-κB alpha (IκBa) through its *N*-terminal RNA-binding domain, resulting in the activation of nuclear factor κ B (NF-κB) [72].

## 5. The Role of hnRNP A1 in Constitutive and Alternative mRNA Splicing

The removal of intronic sequences in the nascent transcript is carried out by the sequential assembly of a large multicomponent ribonucleoprotein complex, the spliceosome, constituted by five core small nuclear ribonucleoproteins (snRNPs, U1, U2, U4, U5, U6) [73,74], whose assembly onto the pre-mRNA requires several auxiliary splicing factors [75,76]. Exons from a primary transcript can be spliced in different arrangements to yield mRNAs that will produce functionally different protein variants [77,78]. The sequencing of the human genome and transcriptome has revealed that over 90% of multi-exon genes are alternatively spliced in tissue-specific and developmentally-regulated manners, providing a major mechanism for the regulation of gene expression [79–81]. 5′ splice sites (5′ ss) and 3′ splice sites (3′ ss) are short, loosely conserved sequences flanking the introns, which are required for splicing, but alone are not sufficient for the proper recognition of exonic and intronic sequences. Additional regulatory elements are classified as either exonic and intronic splicing enhancers (ESE and ISE) or exonic and intronic splicing silencers (ISS and ESS). Positive and negative *cis*-acting sequences are often organized in multipartite control elements. The best-known splicing silencers are dependent on interactions with the hnRNPs of the A/B and H/F sub-families [82], while the best characterized exonic splicing enhancers are purine-rich sequences that recruit members of the serine/arginine-rich (SR) family of splicing activators. SR-dependent ESEs act by recruiting and stabilizing components of the core splicing machinery to nearby splice sites [83]. SR proteins and hnRNPs often play counteracting roles within the same splicing regulatory unit composed by clusters of several, often overlapping, ESSs and ESSs [15,18,84].

A constitutive role for hnRNP A1 in the multi-step process, leading to the catalytic excision of the intervening intron and joining of the adjacent exons, is suggested by comprehensive proteomic analysis of splicing complexes, indicating that this protein participates in all steps of spliceosome assembly [76,85]. Further proof for a role of hnRNP A1 in the activity of the basic splicing machinery is given by recent results showing that it forms a ternary complex with the essential splicing factor, U2AF, and helps the splicing machinery discriminating between cryptic and functional 3′ splice sites [86].

**Figure 2:**
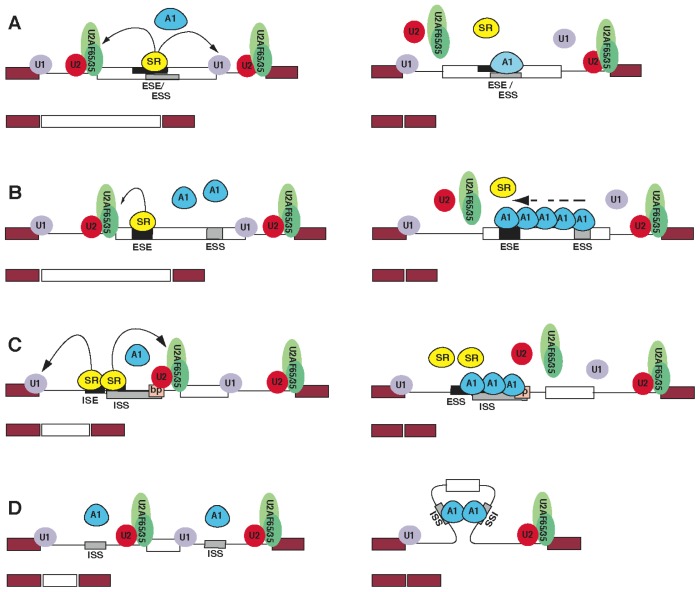
Splicing regulation mechanisms of hnRNP A1. (**A**) Serine/arginine-rich (SR) proteins bound to an exonic splicing enhancer (ESE) promote recruitment of splicing factors to nearby splice sites. Binding of hnRNP A1 to an exonic splicing silencer (ESS) overlapping an ESE displaces the SR proteins and promotes skipping of the exon from the mRNA [18,88–93]; (**B**) hnRNP A1 binding to a high affinity binding site, which functions as an ESS, promotes cooperative binding of other hnRNP A1 molecules along the transcript. This inhibits the binding of SR proteins and other splicing factors and promotes exclusion of the exon from the mRNA [94]; (**C**) Binding of hnRNP A1 to an intronic splicing silencer (ISS) overlapping an SR-dependent intronic splicing enhancer (ISE), or the branch point (bp) displaces SR proteins or the U2 snRNP and inhibits splicing of the downstream exon [18,95,96]; (**D**) The interaction among hnRNP A1 proteins bound to ISS upstream and downstream the alternatively spliced exon promotes looping-out and exclusion of the exon [16,97,98].

hnRNP A1’s role as a modulator of alternative splicing has been widely studied. hnRNP A1 was initially identified as a switch for splicing site selection using model and adenovirus E1A pre-mRNAs [87] and quickly became the most studied splicing repressors in both cellular and viral systems. Experimental observations in several genes (summarized in Table 1) established a key role for hnRNP A1 in a number of cellular mechanisms regulating development and a number of cellular responses to endogenous and exogenous stimuli and disease. Binding of hnRNP A1 to several high-affinity exonic and intronic sequences has been identified as essential for repression of splicing in human and viral genes. Biochemical and structural studies have uncovered several mechanisms utilized by hnRNP A1 to modulate splicing: (i) In some alternatively spliced exons, hnRNP A1-dependent ESSs overlap ESEs bound by SR proteins, which promote spliceosome assembly, and the competition between hnRNP A1 and SR proteins for common binding sites determines the ratio of inclusion/exclusion of the exon (Figure 2A). Examples of this type of regulation are the *HipK3* germline-specific exon [88], the bovine growth hormone exon 5 [89,90], the *c-src* exon N1 [91], the HIV-1 *tat* exon 2 [18] and the *SMN2* exon 7 [92,93]; (ii) In other cases, when hnRNP A1-dependent ESSs and SR-dependent ESEs do not overlap, hnRNP A1 can bind cooperatively along the exon, limiting the access of SR proteins or other splicing factors to their binding sites (Figure 2B). Detailed work on the mechanism of hnRNP A1 cooperative binding showed that, after binding to a high-affinity site, hnRNP A1 spreads preferentially in a 3′ to 5′ direction and can displace other bound proteins from the RNA to repress splicing [94]; (iii) Specific binding sites for hnRNP A/B proteins also exist in introns and may inhibit the binding of the key splicing regulator onto nearby intronic regions. Inclusion of exon 2 and 3 of the *IRF-3* gene is dependent on an ISS binding hnRNP A1 within the first intron of the gene. The hnRNP binding site is located in proximity of a series of ISEs bound by the SR protein, SF2. hnRNP A1 binding is likely to displace SF2 and downregulate splicing of the downstream exons (Figure 2C) [95]. This is similar to the mechanism regulating splicing of HIV-1 *tat* exon 2, where an hnRNP A1-dependent ESS is juxtaposed to an ESE recognized by the SR protein, SC35 [18]. In a different system, the HIV-1 *tat* exon 3′ upstream intron, hnRNP A1 binds an ISS that overlaps a branch point. This is a conserved sequence located upstream of the 3′ splice site that is bound by the U2 snRNP and is required for spliceosome assembly and efficient excision of the intron. hnRNP A1 binding onto the ISS competes with the U2 snRNP recognition of the branch point and inhibits spliceosome assembly [96]. (iv) An alternative “looping out mechanism” has been proposed for the alternative splicing of the *hnRNP A1* exon 7B, which is flanked by multiple binding sites for hnRNP A1. Binding of hnRNP A1 onto the ISSs promotes the skipping of exon 7b. It is postulated that hnRNP A1 molecules bound at both sides of exon 7b can interact through their Gly-rich domain and loop out the intervening exon (Figure 2D) [16,97,98]. hnRNP A1 binding sites are found flanking several alternatively-spliced eukaryotic exons and often co-localize with binding sites for members of the hnRNP H/F protein family. hnRNP A1 and hnRNP H have been shown to have the potential to collaborate to modulate splicing by interacting through their Gly-rich domain [15,28].

Functional and physical interactions with a number of other splicing regulators are also possible and might contribute to the alternative splicing functions of hnRNP A1 [123]. Recent studies on the splicing activities of six hnRNPs (A1, A2/B1, H1, F, U and M) carried out utilizing a genomic approach show that, although the high degree of homology present between hnRNP A1 and A2/B1, hnRNPs A2/B1, H1, F and U act cooperatively to regulate the same set of alternative splicing events, hnRNPs A1 and M frequently influence changes in opposition to the other hnRNP proteins [11]. The growing amount of data obtained through genomic, cellular and molecular approaches indicate that regulation of alternative splicing by hnRNP A1 is pervasive throughout the human transcriptome and utilizes a number of mechanisms in synergy with several other splicing factors.

## 6. hnRNP A1, Telomeres Maintenance and Telomerase Activity

hnRNP A1 also plays a role in DNA metabolism associated with telomeres, contributing to telomere length regulation and maintenance. Telomeres are conserved tandem arrays of repetitive DNA sequences (TTAGGG in vertebrates) that function by interacting with a number of proteins and protecting the ends of chromosomes from being detected as broken DNA by the repair system, thus preventing degradation and fusion [124]. Human telomeres end with a 3′ single-stranded G-rich overhang of 12–300 nucleotides, which invade the homologous double-stranded telomere to create a telomere loop structure (t-loop) with the help of a complex of telomere-bound proteins, termed shelterin [125]. The single-stranded G-rich overhang can also induce G-quadruplex formation [126] and, during development and in some cell types, including cancer and stem cells, provides the substrate required for elongation by telomerase, a ribonucleoprotein that possesses reverse transcriptase activity (TERT), capable of catalyzing the addition of telomere repeats utilizing an internal RNA template [124].

In most human somatic cells, telomeres shorten as cells divide, and failure to maintain telomeres induces cell cycle arrest, senescence and apoptosis. Failure of the mechanisms detecting the shortening of telomeres leads to chromosome instability and may trigger malignant transformation [127]. hnRNP A1 has been shown to bind telomeric sequences and plays a critical role in telomere biogenesis [24] and maintenance by promoting telomerase activity and telomere length extension. *In vitro* depletion of hnRNP A1 from human cell extracts reduces telomerase activity, which is fully recovered upon addition of purified recombinant hnRNP A1 [128]. Similar results have been also achieved in *in vivo* models, showing that telomeres are shorter in an hnRNP A1-deficient murine cell line, and addition of hnRNP A1 restores telomere length [129]. It has been proposed that hnRNP A1 might contribute to the telomerase function by its ability to unwind the G-quadruplex structures of telomeres [128] and, at the same time, bind the RNA component of the telomerase [130] and TERRA, the RNA component of the telomere complex [131]. Recent work also suggests that hnRNP A1 facilitates telomeric end-capping following replication upon phosphorylation by the DNA-PKcs kinase [35,132].

Although most of the studies indicate a positive role in telomere elongation for hnRNP A1, *in vitro* data suggest that the binding of hnRNP A1 to the single-stranded telomeric ends protects such sequences against degradation and, at the same time, inhibits the telomerase activity [133]. The multiple functions assigned to hnRNP A1 in telomere maintenance and elongation indicate that hnRNP A1 might play different roles, depending on its phosphorylation state or the presence of other co-factors.

## 7. mRNA Nuclear Export and hnRNP A1

hnRNP A1 is found to be associated with poly(A)+ RNA in both the nucleus and cytoplasm [134] and is one of the components of the hnRNP complex that accompanies mature transcripts through the nuclear pores [37], and it associates with mRNA export factors [134]. The shuttling properties of hnRNP A1 are dependent on the M9 sequence that functions as both a nuclear export and a nuclear localization signal [135].

*In vitro* and *in vivo* observations suggest a role for hnRNP A1 in the nuclear export of a number of mRNAs. Detailed analysis of mRNA trafficking through the nuclear pores carried out utilizing *dihydrofolate reductase* (*DHFR*) gene transcripts indicates that expression of hnRNP A1 or M9 peptides inhibits mRNA export [136,137], while electron microscopy studies and, more recently, light sheet microscopy showed that hnRNP A1-like proteins bind to the giant Balbiani ring mRNA in Chironomus tentans and accompany this mRNA to the cytoplasm [138,139]. Inhibition of RNA polymerase II in HeLa cells by actinomycin D and the study of transcriptionally inactive mouse embryos also indicate that newly synthesized mRNA acts as an inducer for the nuclear import of hnRNP A1 [37,140]. Together, these data are consistent with the notion that hnRNP A1 plays a role in mRNA export. Nevertheless, data showing that hnRNP A1 antibodies efficiently immunoprecipitate excised introns, but not the spliced transcripts, suggest a role in packaging intronic sequences, which are retained within the nucleus after splicing and, ultimately, degraded [141]. Thus, it is still unclear if hnRNP A1 plays a passive role in mRNA export, a more active one, by packaging excised introns and retaining them in the nucleus, or if it is involved in the export of only specific mRNAs subsets. It is plausible that this protein might exert multiple roles in mRNA nuclear retention and export depending on its phosphorylation state, relative expression levels and interactions with other cellular partners.

## 8. hnRNP A1 Regulates mRNA Translation and Turnover

hnRNP A1 also plays roles in modulating the expression of fully processed mRNAs. AU-rich sequences (AREs), which have been shown to modulate mRNA turnover and translation in a number of mRNAs [142], are specifically bound by hnRNP A1 in the mRNAs coding for *interleukin 2 (IL-2)* and granulocyte-macrophage colony-stimulating factor (GM-CSF), suggesting a role in regulating mRNA stability for this protein [143,144].

hnRNP A1 activities in both cap-dependent and cap-independent translation mechanisms have also been observed. Addition of hnRNP A1 in a rabbit reticulocyte lysate rendered translation cap-dependent, possibly by promoting ribosome binding at the m7G cap, preventing initiation at aberrant translation start sites [145]. In cap-independent translation systems, hnRNP A1 has been shown to bind internal ribosomal entry site (IRESs) sequences, which govern cap-independent translation initiation in cellular and viral mRNAs [146]. The assembly of hnRNP A1 onto IRESs has been shown to enhance IRES-mediated translation of the *human fibroblast growth factor 2 (FGF-2)* mRNA [147], the human rhinovirus (HRV) [148] and, more recently, the transcription factor, *SREBP-1 (sterol-regulatory-element-binding protein 1)* in hepatocytes [149]. Studies on the expression of the *cyclin D1* and *c-myc* mRNAs show that hnRNP A1 constitutively binds and promotes translation from the IRES of both genes, and this activity is negatively modulated by phosphorylation of hnRNP A1 by the Akt kinase [150,151]. Interestingly, in a few other systems, such as the *X-linked inhibitor of apoptosis (XIAP)* mRNA [152] and the human *apoptotic peptidase activating factor 1 (apaf-1)* [148], hnRNP A1 has been shown to inhibit IRES-mediated translation. These, apparently contradictory, data suggest that the role of hnRNP-A1 in translation is dependent on other co-factors, which might modify its activity in different systems.

## 9. hnRNP A1 Regulates miRNA Processing

Given hnRNP A1’s ubiquitous RNA binding properties and its ability to interact with a number of cellular factors, it is not surprising that it has a role in the processing of a number of miRNAs, short non-coding RNAs that act as regulators of gene expression in many different biological processes [153]. miRNAs base-pair with target mRNAs and negatively regulate their expression by two mechanisms: reducing the efficiency of translation and shortening the transcript half-life by recruiting an enzymatic complex called the RNA-induced silencing complex (RISC). The biogenesis of mature miRNAs is a multistep process that initiates in the nucleus. After transcription, the pri-miRNA precursors are cleaved by the RNase III Drosha enzyme and DGCR8, which results in the production of stem loop precursors, termed pre-miRNAs. Following export to the cytoplasm, the pre-miRNAs are processed by the type III ribonuclease, Dicer, resulting in the production of mature miRNAs that are then loaded onto the RISC complex [154].

The crosslinking and immunoprecipitation protocol (CLIP) carried out to search for hnRNP A1 endogenous RNA targets has revealed that this protein binds specifically to human pri-miR-18a [155]. A detailed biochemical analysis revealed that hnRNP A1 facilitates miR-18a production by binding to the terminal loop of its pri-miRNA and induces a relaxation at the stem, creating a more favorable cleavage site for Drosha, thus acting as an auxiliary factor for the processing of the miRNA precursor [155]. It is unclear if this regulatory mechanism is common to other miRNAs; nevertheless, phylogenetic data suggests that a class of miRNAs might be regulated by the interactions with hnRNP A1 and other RNA binding factors [156]. Surprisingly, recent results revealed that hnRNP A1 negatively regulates expression of the human Let-7a, a member of the Let-7 family of miRNAs, which is present in multiple copies in different genomes and plays roles in cancer and pluripotency by targeting several genes [157]. hnRNP A1 binds the conserved terminal loop of pri-let-7a and inhibits its processing by Drosha, thus acting as a negative regulator of miRNA expression [158]. The effect of hnRNP A1 on Let-7a expression is the opposite of that on miR-18a, thus acting as a negative regulator of miRNA expression. Consistent with this observation, the molecular mechanisms regulating these two systems differ. Instead of facilitating pri-Let-7a processing by Drosha, hnRNP A1 binding to the pri-Let-7a loop interferes with the binding of the splicing regulatory protein, KSRP, known to promote Let-7a biogenesis [159]. Therefore, hnRNP A1 and KSRP play antagonistic roles in the post-transcriptional regulation of Let-7a by competing for overlapping binding sites within the conserved loop of pri-Let-7a. While the role of hnRNP A1 in controlling miRNA biogenesis has been studied, little is known of the hnRNP-targeted miRNAs. Studies focused on the isolation and functional characterization of such targets may uncover additional relevant regulatory pathways modulated by hnRNP A1.

## 10. hnRNP A1 Role in Human Disease and Therapy

Given its multiple functions in gene expression and cellular metabolism, hnRNP A1 has been shown to play a key role in human disease. A number of studies utilizing biochemical, cellular, animal and, more recently, genomic approaches have unveiled the roles of hnRNP A1 in genetic deficiencies, cancer development, metastasis, neurodegeneration and replication of viral pathogens.

The relevance in disease of hnRNP A1 is highlighted by its deregulated, usually over-overexpression, in a wide variety of cancers, including breast, colorectal, lung and gliomas [120,160–165], and has been found to promote tumor invasion and to be connected to poor prognosis in hepatocellular carcinoma [166]. Consistent with its role in oncogenesis, siRNA-mediated knockdown of hnRNP A1 in cancer cells results in apoptosis [167], while its expression has antiapoptotic effects, possibly by affecting splicing of caspase*-*2 pre-mRNA [168].

hnRNP A1 plays several key roles in neuronal functions, and since its expression causes drastic changes in RNA metabolism, variation in its abundance contributes to neurodegenerative diseases, such as Alzheimer’s disease (AD), spinal muscular atrophy (SMA), multiple sclerosis (MS), amyotrophic lateral sclerosis (ALS), fronto-temporal lobar degeneration (FTLD), HTLV-I associated myelopathy/tropical spastic paraparesis (HAM/TSP) and hereditary spastic paraparesis (HSP) [169]. Alzheimer disease patients exhibit a reduction of hnRNP A1 expression in the brain, and in a mice model, loss of hnRNP A1 is associated with impaired cognitive function [170]. HnRNP A1 has also been shown to modulate alternative splicing of the APP gene, affecting the generation of toxic Aβ peptide, which accumulates in the amyloid plaques characteristic of the Alzheimer disease brain [113]. The inverse changes in hnRNP A1 levels in tumors and neurodegenerative diseases might point out key differences in the molecular pathologies of such diseases.

An intriguing recent report shows that hnRNP A1 might function as a prion-like protein, inducing a series of pathogenesis through a unique and novel mechanism [100]. Families with inherited degeneration affecting muscle, brain, motor neuron, bone and amyotrophic lateral sclerosis carry distinctive mutations within the hnRNP A1 Gly-rich domain. The ability of hnRNP A1 to multimerize is enhanced by the mutations detected in patients. Furthermore, the mutated protein can increase the ability of the wild-type hnRNP A1 to multimerize into fibrils and, in an animal model, the formation of cytoplasmic inclusion. Thus, hnRNP A1 could be an important component of human proteinopathies.

Although many human pathologies and physiological responses correlate with a change in the expression level of hnRNP A1, little is known of the mechanisms regulating the abundance of this protein. Analysis of the cellular targets of the RNA binding protein, Quaking (Qk), which is required for myelin formation and associated with psychiatric disease, showed that the 3′ UTR of the hnRNP A1 messenger contains a binding site for this protein [171]. Qk regulates the overall level of hnRNP A1 by stabilizing its mRNA. Genome-wide analyses showed that hnRNP A1 contributes to Qk control of myelin gene expression, thus consolidating a role for hnRNP A1 in neural pathogenesis.

hnRNP A1 has also been shown to participate in the mechanisms regulating the gene expression and replication of a number of viruses: human rhinovirus [148], Enterovirus 71 [172], Sindbis virus [173], hepatitis C virus [174], human papilloma virus [175,176] and, more extensively, HIV-1 [177,178]. The role played by hnRNP A1 in HIV-1 gene expression has been widely studied and has been utilized as a model to elucidate the mechanisms regulating cellular mRNA processing. Biochemical, structural and cellular approaches have identified multiple high-affinity hnRNP A1 binding sites within the viral transcript. The interaction of hnRNP A1 with the viral transcript and the interplay with other cellular splicing factors tightly regulate a series of splicing events that generate over 40 mRNAs from the single viral transcript. Furthermore, hnRNP A1 acts to upregulate IRES-mediated translation initiation of the viral mRNA [179] and possibly stimulates the export through the nuclear pore complex [180].

The development of reagents and compounds that specifically target the activity of hnRNP A1 might be of great help in the development of novel therapeutics for a number of diseases and pathogens. Targeting the splicing activity of hnRNP A1 has been recently exploited in the treatment of spinal muscular atrophy (SMA), a neurodegenerative disease in which cellular death of spinal cord motor neurons results in system-wide muscle atrophy [181]. SMA is caused by loss of the Survival Motor Neuron 1 (SMN) protein, due to mutation within the *SMN1* gene. *SMN2* is a paralogous gene that produces a low level of the SMN protein, due to the presence of a mutation, which creates a novel hnRNP A1 binding site, which, in turn, induces alternative splicing of an exon, generating an mRNA coding for a defective SMN protein [92]. Recent work in cellular and animal models showed that antisense oligonucleotide analogs masking the hnRNP A1-dependent splicing regulatory sequence in *SMN2* correct the splicing event and induce the production of functional SMN proteins [182–185].

A different approach has been recently utilized to inhibit proliferation and migration of a hepatoma cell line. hnRNP A1 is highly expressed in liver cancer tissues compared with either para-cancer or benign controls. Expression of an hnRNP A1-specific single-stranded DNA aptamer blocked hnRNP A1 activity and showed a stronger inhibitory effect on the proliferation of cultured hepatoma cells than hnRNP A1-specific small interfering RNAs [186].

## 11. Conclusions

hnRNP A1 is a multifunctional protein with unique properties that exerts its activities on a wide range of cellular processes. This protein regulates several aspects of mRNA biogenesis, such as transcription, constitutive and alternative splicing, nuclear export and turnover and, at the same time, influences the translation and telomerase machineries. While hnRNP A1 appears to have a constitutive role in some of those processes, it also exerts a specific regulatory function on a number of genes with key functions in health and disease. Intriguing new therapeutic approaches aimed at regulating the expression and functions of hnRNP A1 on specific target mRNAs are being developed. Given the multifunctional nature of this protein, it is difficult to predict the specificity and efficiency of putative new drugs aimed at altering its expression patterns and activities. Nevertheless, novel compounds aimed at masking hnRNP A1 binding sites onto specific substrates are showing promising results.

The identification of the other cellular factors that functionally and physically interact with this protein in a spatial and temporal manner will be of great help to better understand the hnRNP A1 roles in multiple aspects of gene regulation. Genomic and proteomic tools will be essential in developing assays aimed at the identification of the interacting molecules and the functional mapping of hnRNP A1 cellular targets.

## Figures and Tables

**Table 1:** hnRNP A1 role in alternative splicing. 3′ ss, 3′ splice site.

Gene	Organism	Spicing event	Reference
*Medium-chain acyl-CoA dehydrogenase (MCAD)*	Human	Exon 11 skipping	[99]
*Myelin-associated glycoprotein (MAG)*	Human	Exon 12 skipping	[100]
*Interferon regulatory factor-3 (IRF-3)*	Human	Exons 2 and 3 skipping	[95]
*TNF Receptor Superfamily Member 6 (Fas)*	Human	Exon 6 skipping	[101]
*Ras-related C3 botulinum toxin substrate 1 (Rac1)*	Human	Exon 3b skipping	[102]
*Insulin receptor gene (INSR)*	Human	Exon 11 skipping	[103]
*Breast cancer 1 (BRCA1)*	Human	Exon 18 skipping	[104]
*Breast cancer 1 (BRCA1)*	Human	Exon 6 skipping	[105]
*Homeodomain interacting protein kinase 3 (HIPK3)*	Human	Testis-specific Exon skipping	[88]
*Bovine growth hormone (BGH)*	Bovine	Exon 5 skipping	[89,90,106]
*Survival of Motor Neuron 2, (SMN2)*	Human	Exon 7 skipping	[92,107–110]
*Fibroblast growth factor receptor 2 (FGFR2)*	Human	K-SAM exon skipping	[111,112]
*Amyloid precursor protein (APP)*	Human	Exon 7 and 8 skipping	[113]
*Dystrophin*	Human	Exon 31 skipping	[114]
*β-tropomyosin*	Chicken	Exon 6B skipping	[115]
*pX region*	Human T-cell leukemia virus type 1 (HTLV-1)	Exon skipping	[116]
*V-Ha-ras Harvey rat sarcoma viral oncogene homolog (C–H-ras)*	Human	Exon IDX skipping	[117]
*Proto-oncogene tyrosine-protein kinase Src (c-SRC)*	Human	Exon N1 skipping	[91]
*Trans-activator of transcription (Tat)*	Human immunodeficiency virus type 1 (HIV-1)	Exon 3 3′ ss repression	[48,51,94,96,118]
*Trans-activator of transcription (Tat)*	Human immunodeficiency virus type 1 (HIV-1)	Exon 2 3′ ss repression	[18,49]
*Carcinoembryonic antigen-related cell adhesion molecule-1 (CEACAM1)*	Human	Exon 7 skipping	[119]
*heterogeneous ribonucleoprotein A1 (hnRNP A1)*	Human	Exon 7B skipping	[16,97,98]
*Pyruvate kinase (PKM)*	Human	Exon 9 skipping	[120]
*Viral protein R (VPR)*	Human immunodeficiency virus type 1 (HIV-1)	Repression 3′ splice site A2	[121]
*E6/E7*	Human papillomavirus type-16 (HPV-16)	E6 exon skipping	[122]
